# Concentrated LiODFB Electrolyte for Lithium Metal Batteries

**DOI:** 10.3389/fchem.2019.00494

**Published:** 2019-07-18

**Authors:** Juan Yu, Na Gao, Jiaxin Peng, Nani Ma, Xiaoyan Liu, Chao Shen, Keyu Xie, Zhao Fang

**Affiliations:** ^1^School of Metallurgical Engineering, Xi'an University of Architecture and Technology, Xi'an, China; ^2^State Key Laboratory of Solidification Processing, Center for Nano Energy Materials, School of Materials Science and Engineering, Northwestern Polytechnical University and Shaanxi Joint Laboratory of Graphene (NPU), Xi'an, China

**Keywords:** Li-metal batteries, concentrated electrolyte, LiODFB, high temperature, dendrites free

## Abstract

Nowadays, lithium (Li) metal batteries arouse widespread concerns due to its ultrahigh specific capacity (3,860 mAh g^−1^). However, the growth of Li dendrites has always limited their industrial development. In this paper, the use of concentrated electrolyte with lithium difluoro(oxalate)borate (LiODFB) salt in 1, 2-dimethoxyethane (DME) enables the good cycling of a Li metal anode at high Coulombic efficiency (up to 98.1%) without dendrite growth. Furthermore, a Li/Li cell can be cycled at 1 mA cm^−2^ for over 3,000 h. Besides, compared to conventional LiPF_6_-carbonate electrolyte, Li/LiFePO_4_ cells with 4 M LiODFB-DME exhibit superior electrochemical performances, especially at high temperature (65°C). These outstanding performances can be certified to the increased availability of Li^+^ concentration and the merits of LiODFB salt. We believe that the concentrated LiODFB electrolyte is help to enable practical applications for Li metal anode in rechargeable batteries.

## Introduction

In the past several decades, Li-ion batteries have played successful role in the consumable electronic device market (Etacheri et al., 2011; Goodenough and Kim, 2014). However, the limited specific capacity of the graphite anode limits its wider applications. The theoretical capacity of graphite is only 372 mAh g^−1^, so it is difficult to achieve the urgent need for high-energy density batteries to adapt to the electrical miniaturization trend (Placke et al., 2017). Based on this reason, the Li metal batteries (LMBs) have attracted wide attention because of its high theoretical specific capacity of 3,860 mAh g^−1^, small density of 0.534 g cm^−3^ and the relatively negative electrochemical potential (−3.040 V vs. Li/Li^+^) (Zhamu et al., 2012). However, the shortcomings of LMBs are also a headache, such as unsatisfied Coulombic efficiency (CE) and dendritic Li growth. In addition, poor cycle performance caused by Li dendrites seriously affected the commercial development of LMBs.

Significant efforts have been made in the past to solve these problems, including prepare the current collector (Yang et al., 2015; Liang et al., 2016; Zhang et al., 2016; Shi et al., 2017), *ex-situ* protective coating of Li anode (Thompson et al., 2011; Kozen et al., 2015), introduce the battery intermediate protective layer (Liang et al., 2015; Wu et al., 2015; Cheng et al., 2016; Xie et al., 2016, 2017), and electrolyte modification. Electrolyte, which occupies 15% of the weight and about 32% of the volume of the entire battery, is a key part of a battery (Cekic-Laskovic et al., 2017). Therefore, the study of electrolyte on the development of Li metal secondary battery is of great significance. Meanwhile, compared to other modification methods, electrolyte modification is more convenient and cost-effective. Till now, electrolyte research progress more or less has been made to address the dendrite problem. Nevertheless, the compatibility of these electrolytes with cathode has usually been overlooked. For example, some film-forming electrolyte additives, such as vinylene carbonate (VC) and fluoroethylene carbonate (FEC) are helpful to change Li dendrite formation nature (Aurbach et al., 2003; Jung et al., 2013; Webb et al., 2014; Qian et al., 2016; Pritzl et al., 2017; Xu et al., 2017), but the cell's internal resistance is greatly increased, particularly after long-term cycling, and thus limits their wide applications.

Recently, the concept of high concentrated electrolyte gets into the eyes of people. Suo et al. reported a new class of non-aqueous liquid “Solvent-in-Salt” electrolytes and applied them in Li-S batteries. It is demonstrated that the use of “Solvent-in-Salt” electrolyte inhibits the dissolution of polysulphide and protects metallic Li anodes against the formation of Li dendrites, effectively (Suo et al., 2013). A significant breakthrough has been achieved by Qian et al. (2015). They found that, with 4 M lithium bis(fluorosulfonyl)imide (LiFSI) in DME as the electrolyte, a Li/Li cell can be cycled at for more than 6,000 cycles. Meanwhile, a Cu/Li cell can be cycled for more than 1,000 cycles with an average CE of 98.4%, without dendrite growth. Nevertheless, the compatibility of the electrolyte with the cathode materials has not been investigated.

Generally, LiFSI has inherent characteristics of corrosion of aluminum (Al) foil and other metal parts in the battery (Abouimrane et al., 2009; Li et al., 2011). To solve this problem, Park et al. found that lithium borate salts are the ideal additives as corrosion inhibitors in LiFSI electrolytes. The inhibition ability of Al is revealed to be in the following order: lithium oxalyldifluoroborate (LiODFB) > lithium tetrafluoroborate (LiBF_4_) > lithium hexafluorophosphate (LiPF_6_) > lithium bis(oxalato)borate (LiBOB) (Park et al., 2015). Noticed that LiODFB is considered to combine the half structures of LiBOB and LiBF_4_, and thus, it combines the advantages of LiBOB and LiBF_4_ (Zhang, 2006). The main advantages of this salt is given by low viscosity, high ionic conductivity, good film-forming, high temperature performance, good compatibility with the positive electrode, passivation of Li foil and so on (Liu et al., 2007; Zugmann et al., 2011; Wu et al., 2012; Zhou et al., 2012).

However, it should be noted that previous studies (Zhang et al., 2010; Li et al., 2015; Zhou et al., 2016; Bian et al., 2017) only focused on the effect of LiODFB as an additive or auxiliary salt on battery performances. So far, high concentration of LiODFB single salt in ether solvent for LMBs has not been systematically reported before (Zhang et al., 2015; Poyraz et al., 2019; Yamada et al., 2019). Thus, in this work, we first employed the concentrated electrolyte based on LiODFB in LMBs. Due to its increased availability of Li^+^ concentration, a highly uniform and stable solid electrolyte interface (SEI) film was formed on Li metal anode without any dendrite. Meanwhile, the compatibility of concentrated LiODFB electrolyte and LiFePO_4_ cathode was first investigated by the electrochemical performance of Li/LiFePO_4_ cell at room temperature and high temperature. We found that 4 M LiODFB-DME significantly improve electrochemical performance of LiFePO_4_ cathode, compared to LiPF_6_ electrolyte. In particular, at high temperature, the improvement is much greater. In a sense, our work shows concentrated LiODFB electrolyte may have great potential for LMBs.

## Experimental Section

### Electrolytes and Electrode Preparation

The LiPF_6_ dissolved in ethylene carbonate (EC), ethyl methyl carbonate (EMC), dimethyl carbonate (DMC) with a volume ratio of 1:1:1 was formed 1.0 M LiPF_6_ electrolyte and used as the blank electrolyte. LiODFB was purchased from Suzhou Fluolyte Co., Ltd., China. LiODFB was dissolved in dimethyl ether (DME) and formed 4 M LiODFB-DME in an Ar-filled glove box (H_2_O < 0.1 ppm, O_2_ < 0.1 ppm). The electrolytes were stirred to maintain homogeneity, then sealed and stored in the glove box.

The LiFePO_4_ cathodes were prepared by mixing 80 wt.% LiFePO_4_ (Aladdin Co., Ltd. China), 10 wt.% conductive carbon, and 10 wt.% polyvinylidene (PVDF, Solef) binder in N-methylpyrrolidone (Kelong, Chengdu). The mixed slurry was coated onto Al foil and then dried overnight at 110°C under vacuum. Disk-shaped electrodes with a diameter of 13 mm were then punched from the foil (the active material loading was 1.52 mg cm^−2^). CR2016 coin-type cells were assembled in an Ar-filled glove box with an LiFePO_4_ cathode working electrode, a Li foil counter electrode, and a polypropylene (Celgard 2400) separator; 45 mL of electrolyte was then injected into the cells. Finally, the assembled coin cells were sealed for further tests. Li/Li and Li/Cu cells with different electrolyte solutions and the above-mentioned separator were assembled for further tests.

### Electrochemical Measurements

The assembled Li/LiFePO_4_ coin cells were left to stand for 10 h before galvanostatic precycling for 200 cycles at 1°C (1°C = 170 mAh g^−1^) between 2.5 and 4.2 V at 25°C using the Land battery test system (Wuhan LANHE Electronics, China). Rate-performance tests were conducted by changing the rate (0.2, 0.5, 1.0, 2.0, 5.0, and 0.2°C) every 10 cycles. Cyclic voltammetry (CV) and electrochemical impedance spectroscopy (EIS) measurements were carried out using a SI-1287 Electrochemical System (Transmission Precision Measurement Company, UK); the CV tests were performed between 2.5 and 4.2 V at a scan rate of 0.1 mV s^−1^ and the EIS tests were performed over 100 kHz to 10 mHz range with an amplitude of 10 mV before and after rate tests. Then the assembled cells were galvanostatic precycled at 65°C.

The current density for the Li metal plating/stripping was set to 1.0 mA cm^−2^ using a Land battery testing station at room temperature. And the deposition time was 1.5 h. The effective area of the Cu foil for Li deposition was 2.11 cm^2^. The current density was 1.0 mA cm^−2^ from 0.01 to 1.00 V. Li/Li symmetric cells were assembled with Li metal used as the working and counter electrodes. The batteries were tested in a Tenney JR environmental chamber to ensure the stable temperature during long-term cycling process.

### Morphological Characterizations

Scanning electron microscopy (SEM) (FE-SEM, LEO 1530) was employed to observe the surface topography of the LiFePO_4_ electrode and Cu foil at different cycling stages at an accelerating voltage of 5 kV. The XPS is tested to further study the composition of SEI film on the Cu foil by the Thermo Electron model K-Alpha surface analysis system. The electrodes retrieved from the cells were thoroughly rinsed in high purity dimethyl carbonate (DMC) solvent for three times and dried under vacuum before transferring to the observation chamber.

## Experimental Results

### Li Metal Deposition Morphology

**Figure 1:**
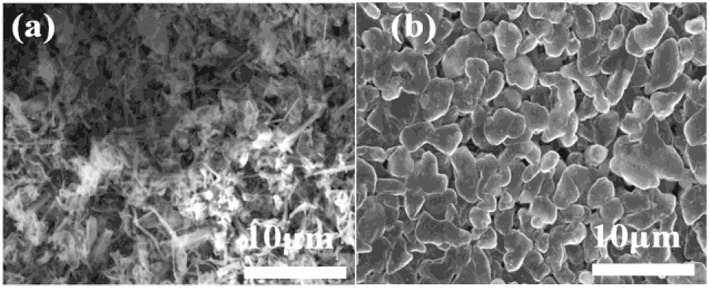
SEM images of the morphologies of Li after plating on Cu substrates in different electrolytes. **(a)** 1 M LiPF_6_ electrolyte; **(b)** 4 M LiODFB-DME. The current density was 1.0 mA cm^−2^ and the deposition time was 1.5 h.

The morphologies of Li deposition in both electrolytes are evaluated by using coin-type Cu/Li cells. The surface morphologies of Li electrodes cycled after 1.5 h in different electrolytes is shown in Figure 1. In conventional carbonate based electrolyte, significant dendrite morphology can be observed (Figure 1a). It can easily pierce most conventional separators, causing the direct contact of the positive and negative electrodes to form a short circuit, and lead to serious security risks. On the contrary, Li deposit from the 4 M LiODFB-DME electrolyte exhibits smooth solid particle morphology (Figure 1b), which dramatically inhibits the growth of Li dendrite. Based on the Li metal deposition morphologies, it is reasonable to conclude that the 4 M LiODFB-DME has good negative electrode stability. The SEI film formed in the high concentration electrolyte has high ionic conductivity, which makes a uniform and stable SEI film formed. The uniform and stable SEI film can suppress the growth of Li dendrite.

### Li Metal Plating/Stripping Cycling Stability

**Figure 2:**
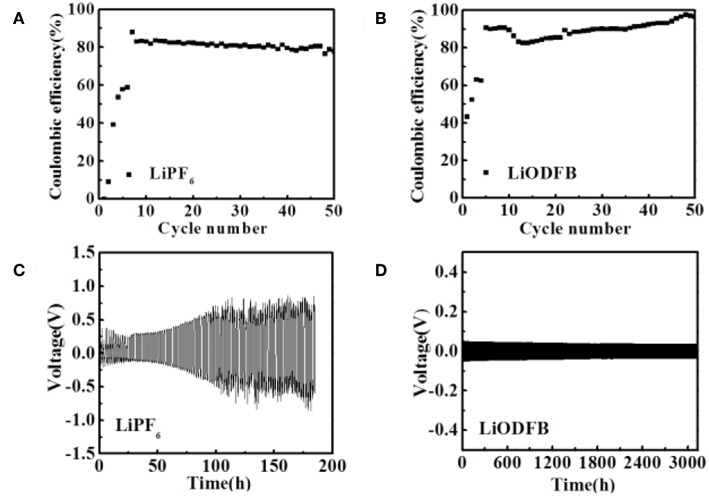
Electrochemical performances of Li/Cu cell **(A,B)** and Li/Li cells **(C,D)** with two electrolytes at a current density of 1.0 mA cm^−2^. **(A,C)** the 1 M LiPF_6_ electrolyte; **(B,D)** the 4 M LiODFB-DME.

The impedance evolution of the coin-type Cu/Li cells after 0.5 h and 1.5 h are depicted in Figure S1. In the Nyquist plots, the intercept with the abscissa represents the ohmic resistance, particularly the electrolyte resistance in the cell. A significant difference in the impedance between the two electrolytes is observed. After 0.5 and 1.5 h of deposition process, the commercial electrolyte exhibits relatively higher electrode impedance Figure S1A. At the same time, 4 M LiODFB-DME electrolyte shows small impedance Figure S1B. The most possible reason for the small impedance is attributed to the formation of the stable SEI film, which is consistent with the results of a dense surface morphology in Figure 1b. Polarization measurements of the Li/Li cells were conducted to understand the effect of different electrolytes on stabling the interface between the Li metal and electrolyte. During this experiment, a constant deposition/dissolution current density of 1.0 mA cm^−2^ was passed through the Li/Li cells for 3,000 h at room temperature. The 1 M LiPF_6_ electrolyte shows the great over-voltage and an ascending tendency with time (Figure 2), which is consistent with previous report (Webb et al., 2014). Surprisingly, the 4 M LiODFB-DME electrolyte appears excellent Li deposition/dissolution performance with the stable and low over-voltage. Polarization is lower than 1 M LiPF_6_ electrolyte. Besides, influenced by high viscosity, on the one hand, it possibly increases the pressure from the electrolyte to push back growing dendrites, resulting in a more uniform deposition on the surface of the anode. On the other hand, high viscosity limits anion convection near deposition area, which is also helpful to deposit uniformly. All of the above results confirm that the high concentration of LiODFB electrolyte in ether solvent can ensure the cycle stability in the negative.

## Discussion

### Discussion for the Possible Function Mechanism

**Figure 3:**
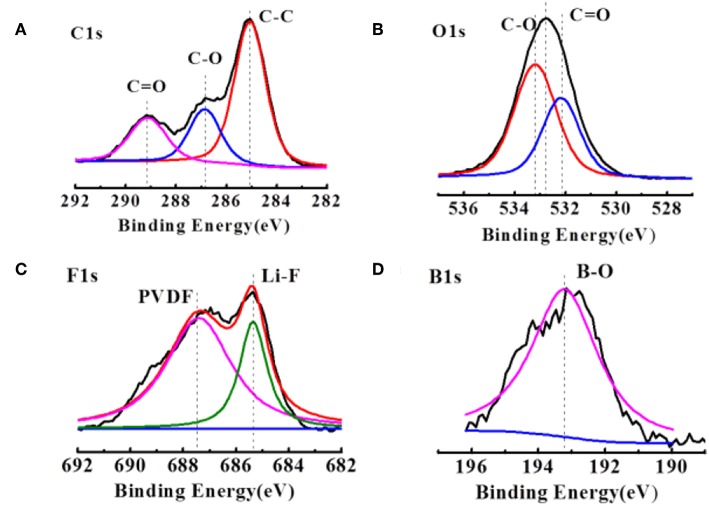
XPS patterns of **(A,B)** the cycled Li anodes in the LiODFB electrolyte. **(C,D)** The cycled electrodes were disassembled from the Li/Li cells after 1.5 h.

**Figure 4:**
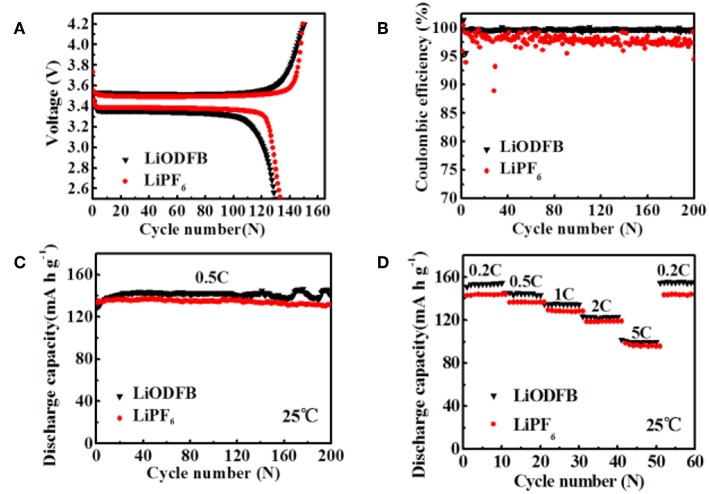
**(A)** first charge/discharge profiles the Li/LiFePO_4_ cells, **(B)** CE of Li/LiFePO_4_ cells; **(C,D)** cycle performance of Li/LiFePO_4_ cells at 25°C with 4 M LiODFB-DME and 1 M LiPF_6_, **(D)** rate performances of Li/LiFePO_4_ cells at 25°C with 4 M LiODFB-DME and 1 M LiPF_6_.

Figure 3A compares the C1s spectra of the delithiated graphite electrodes extracted from the cell after several cycles. The dominant peak at 284.6 eV originates mainly from the sp^2^ hybridized graphite, but also includes contributions from conductive carbon added into the electrode composites. The broad feature at 286.6 eV is assigned mainly to the C atoms of the C-O-C groups in the poly(ethylene oxide), PEO, which is formed upon solvent polymerization. The formation of PEO is also confirmed by the peak at 533.6 eV in the O1s XPS spectra in Figure 3B. The rather broad peaks at 288.6 eV in Figure 4A are assigned to carbon in C = O groups in organic Li alkyl carbonates (ROCO_2_Li) and Li carbonates (Li_2_CO_3_), respectively. The corresponding peak of the oxygen atoms in these groups is detected in the O1s spectra (Figure 3C) at 531.8 eV. Figure 3C, the F1s peak at 687.5 eV are assigned with PVDF, while the peak at 684.2 eV corresponding to LiF is much stronger, agreeing with the inorganic inner layer of SEI. However, there is almost no difference in contrast to the case of the commercial electrolyte in the C1s, O1s, and F1s spectra. The difference appears at F1s spectra. The B1s peaks at 193.8 eV and 192.6 eV (Figure 3D) indicates the presence of LiODFB and material with B–O, respectively. And no such peak in commercial electrolyte.

This result indicates that the B-O bond is involved in the formation of the film, making the SEI film is much denser (Xu et al., 2016). It is consistent with the results of a dense surface morphology in Figure 1b.

### The Electrochemical Performances of LiFePO_4_ Batteries

The assembled Li/LiFePO_4_ coin cells are used to compare the cycling and rate stability using 4 M LiODFB-DME electrolyte and 1 M LiPF_6_ electrolyte at room temperature in this part. Figure 4A shows the first charge/discharge profiles of the cells in different electrolytes at the 0.2°C current rate (1°C = 170 mAh g^−1^). The cells are initially activated at 0.2°C for three cycles and then cycled at 0.5°C. The cells display similar voltage curves, but the cell using the 4 M LiODFB-DME electrolyte exhibits slightly smaller discharge capacities of 129 mAh g^−1^, while the initial discharge capacity of 1 M LiPF_6_ is 133 mAh g^−1^. The result is directly related to the lower ionic conductivity of 4 M LiODFB-DME electrolyte compared to LiPF_6_-based electrolyte. The long-term cycling performances of Li/LiFePO_4_ cells are depicted in Figure 4C. There is no obvious difference after 200 cycles, and 4 M LiODFB-DME electrolyte shows little superiority compared to commercial LiPF_6_-based electrolyte. However, LiODFB electrolyte shows excellent CE of 99% over 200 cycles; while the cell with the LiPF_6_ is only 89% after 200 cycles (Figure 4B). It shows that LiODFB salt greatly improves the cycling stability, but the delivered capacity might have been hindered by the electrolyte's undesirable ionic conductivity. Figure 4D presents the rate capability of Li/LiFePO_4_ cell in 1 M LiPF_6_ and 4 M LiODFB-DME electrolytes. Discharge capacity of cells with 4 M LiODFB-DME are 154.8 mAh g^−1^ at 0.2°C, 143.3 mAh g^−1^ at 0.5°C, 134.4 mAh g^−1^ at 1.0°C, 123.1 mAh g^−1^ at 2.0°C, and 99.5 mAh g^−1^ at 5.0°C. As comparison, discharge capacity of cells with 1 M LiPF_6_ are 143.9 mAh g^−1^ at 0.2°C, 136.7 mAh g^−1^ at 0.5°C, 128.3 mAh g^−1^ at 1.0°C, 118.9 mAh g^−1^ at 2.0°C, and 95.8 mAh g^−1^ at 5.0°C. Voltage profiles of both cells are also very similar Figure S2. It seems that discharged capacity at different cycling rates of cells with 4 M LiODFB-DME electrolyte are slightly higher than cells with 1 M LiPF_6_ electrolyte. The outstanding thermal stability and water stability LiODFB leads to very weak side reactions and the dissolution of iron is suppressed. These are the reasons of better cycling performance of cells in 4 M LiODFB-DME electrolyte. All in all, the electrochemical performances of the two electrolytes capacity reach a considerable level at room temperature. These also can be confirmed on the impedance test results in Figure S2.

**Figure 5:**
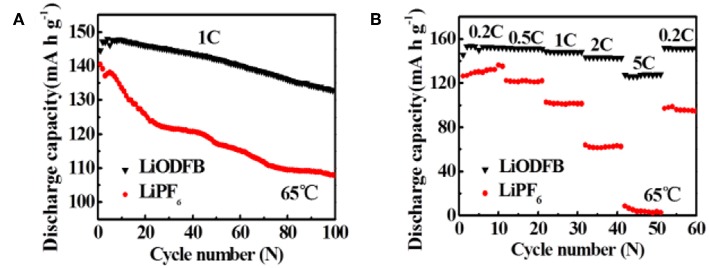
**(A)** cycle performance of the Li/LiFePO_4_ cells at 65°C with 4 M LiODFB-DME and 1 M LiPF_6_, **(B)** rate performance of Li/LiFePO_4_ cells at 65°C with 4 M LiODFB-DME and 1 M LiPF_6_.

Furthermore, the cycling performances of Li/LiFePO_4_ cells at 65°C are assessed in both electrolytes (Figure 5A). In Figure 5A, when the cell is working in LiPF_6_ electrolyte, the capacity decay rapidly after cycles. We can discover it only has capacity retention of 79.3% after 100 cycles, which is consistent with the result reported by ZHANG et al. As a comparison, excellent cycle stability is observed when using 4 M LiODFB-DME electrolyte, with high capacity retention of 92.5% after 100 cycles. The rate capability of Li/LiFePO_4_ at 65°C in different electrolytes is obtained in Figure 5B. Much larger discharge capacities are showed at higher rates for the 4 M LiODFB-DME. For example, the LiPF_6_ electrolyte shows discharge capacities of 101.1 and 62.5 mAh g^−1^ at the 1.0 and 2.0°C rates, respectively, even the capacities of cell is almost close to zero at 5.0°C; While these values are increased to 148.1, 142.8, 127.7 mAh g^−1^ at the 1.0, 2.0, and 5.0°C rates for the LiODFB electrolyte. This demonstrates that LiODFB not only improve the cycling stability but also improve the rate capability in high temperature. It seems that the better thermal stability of LiODFB salts plays an important role in improving the high-temperature resilience of LiFePO_4_ electrode.

**Figure 6:**
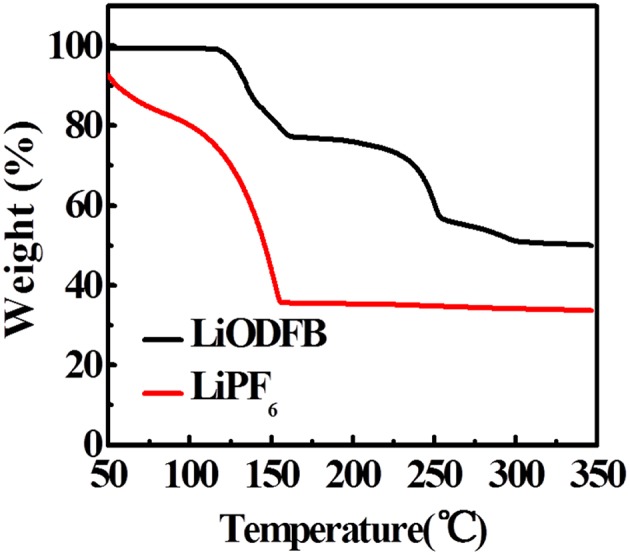
TGA curves of the fully-charged LiFePO_4_ electrode cycled in LiODFB and LiPF_6_ electrolytes.

We used TGA to study the thermal stability of fully charged electrodes in electrolytes (Figure 6). Evaporation of the adsorbed organic solvent appears as their weight loss before 150°C in 4 M LiODFB-DME electrolyte. As the heating progressed, a significant weight loss begins to appear at about 250°C, which originates from the decomposition of the SEI film. In contrast, the large-scale weight decay starts at 50°C in 1 M LiPF_6_, and the mass attenuation has reached a minimum at 150°C, proving that the SEI film has completely broken before 150°C. It shows that 4 M LiODFB-DME electrolyte produces a more stable SEI film and establishes a more gentle interface between the electrode and the electrolyte. So the fully delithiated electrode is protected from directly contacting with the electrolyte. Therefore, the LiODFB electrolyte improves the thermal stability of the highly oxidized charged electrode.

### SEM Micrographs of LiFePO_4_ Electrodes

**Figure 7:**
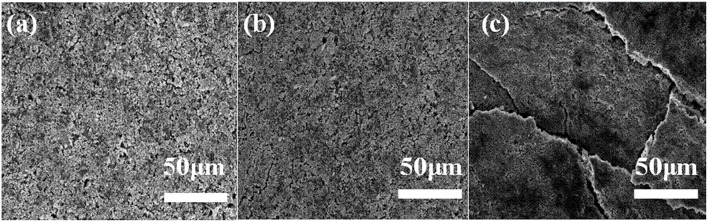
SEM images for pristine cathode **(a)** cathodes after 100 cycles at 65°C with **(b)** the 4 M LiODFB-DME; **(c)** the 1 M LiPF_6_.

SEM analysis is performed to illustrate electrode images before and after 100 cycles in both electrolytes at 65°C. The LiFePO_4_ electrode after long-term cycling in 4 M LiODFB-DME electrolyte (Figure 7b) shows a similar morphology to the pristine LiFePO_4_ electrode (Figure 7a). In comparison, the electrode is severely eroded in 1 M LiPF_6_ electrolyte after 100 cycles at 65°C in Figure 7c, and we can found that the LiFePO_4_ electrode surface in 1 M LiPF_6_ electrolyte displays some cracks among the particles. It is result from the poor thermal stability of the 1 M LiPF_6_ electrolyte. The decomposition of LiPF_6_ in high temperature causes serious capacity decay rapidly, resulting in very poor electrochemical performance, as shown in Figure 5A. The higher the current density, this effect is more serious in Figure 5B. These SEM observations confirm that 4 M LiODFB-DME electrolyte has excellent compatibility with LiFePO_4_ cathodes during battery cycling at high temperature Figure S3 shows the EDS layered image of LiFePO_4_ electrode after 100 cycles at high magnification. The surface of the LiFePO_4_ electrode consists of five main elements: O, P, F, and Fe. The Fe and P elements are mainly derived from LiFePO_4_ electrode and the O, F, and B elements are derived from LiODFB lithium salt. It is proved that the B element from the LiODFB-based electrolyte participates the formation of the surface film of LiFePO_4_ cathode. And the participation of the B elements makes the surface film of LiFePO_4_ cathode smooth and compact. All in all, the LiODFB lithium salt protects the cathode. In addition, the SEM images of the morphologies of Al foils is shown in Figure S4. The surface of the Al foils had a large difference. The concentrated electrolytes suppress the Al corrosion more effectively.

## Conclusions

In this work, we first developed a concentrated electrolyte based on LiODFB in ether solvents for LMBs. Based on 4 M LiODFB-DME electrolyte, Li was deposited on the copper foil and its surface showed smooth solid particle morphology without any dendrites. For the Li/LiFePO_4_ cells, it is demonstrated that 4 M LiODFB-DME electrolyte exhibit good electrochemical performance, especially at the elevated temperature. Li/LiFePO_4_ cells using 4 M LiODFB-DME electrolyte show a good capacity retention (92.5%) at elevated temperature (65°C), which is much higher than that of 1 M LiPF_6_ electrolyte (79.2%). This research offers the possibility of rapid industrialization of LMBs.

## Data Availability

The raw data supporting the conclusions of this manuscript will be made available by the authors, without undue reservation, to any qualified researcher.

## Author Contributions

ZF and JY conceived and designed the experiments. NG, JP, XL, and NM performed the experiments. NG, CS, and XL analyzed the data. NG and NM wrote the manuscript. KX designed the scheme. All authors reviewed the manuscript and approved the final version.

### Conflict of Interest Statement

The authors declare that the research was conducted in the absence of any commercial or financial relationships that could be construed as a potential conflict of interest.
