# Supplementary material for: Concentrated LiODFB Electrolyte for Lithium Metal Batteries
Source: Front Chem. 2019 Jul 18;7:494. doi: 10.3389/fchem.2019.00494 (PMC6657587; doi:10.3389/fchem.2019.00494)
Supplement: Supplementary file 1 [file Image_1.pdf]

Supplementary Material

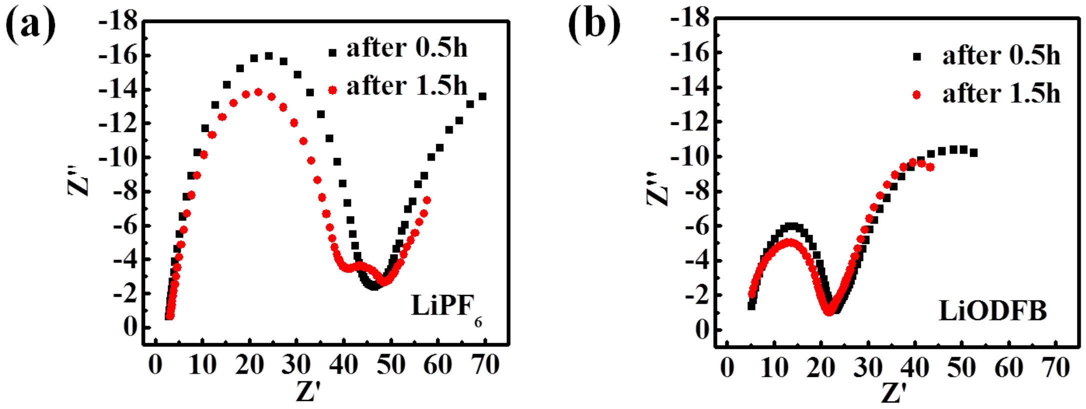

Figure S1 The impedance evolution of the coin-type Cu/Li cells after 0.5 h and 1.5 h. (a) 1 M $LiPF_6$; (b) 4 M LiODFB-DME.

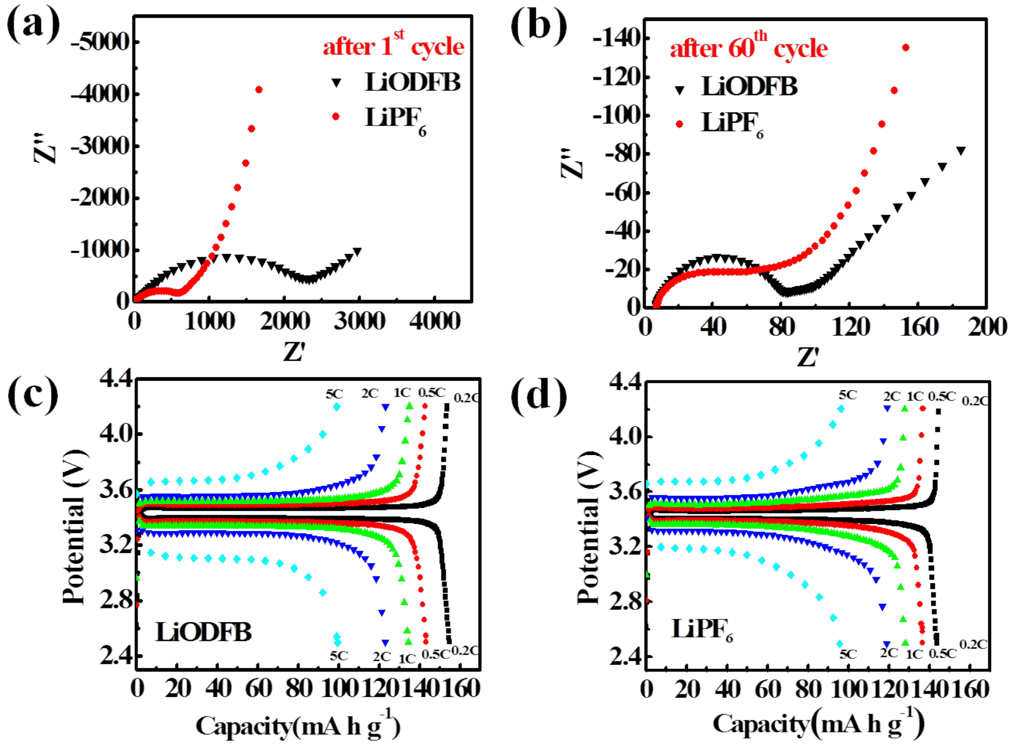

Figure S2 The impedance evolution and voltage profiles of the the Li/LiFePO4 cells (a) after 1st cycle and (b) after 60th cycles with (c) 4 M LiODFB-DME and (d) 1 M $LiPF_6$.

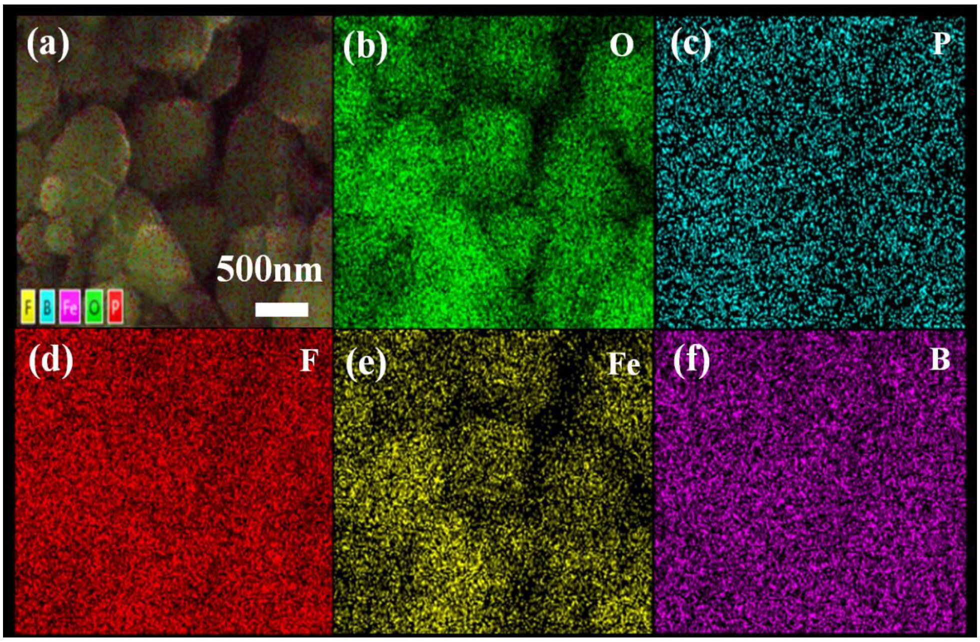

Figure S3 High Resolution Scan EDS Layered Images of LiFePO4 cathode after 100th cycles in 4M LiODFB-DME and the corresponding element mapping of （b） O; （c） N; （d） F; （e） Fe; （f） B

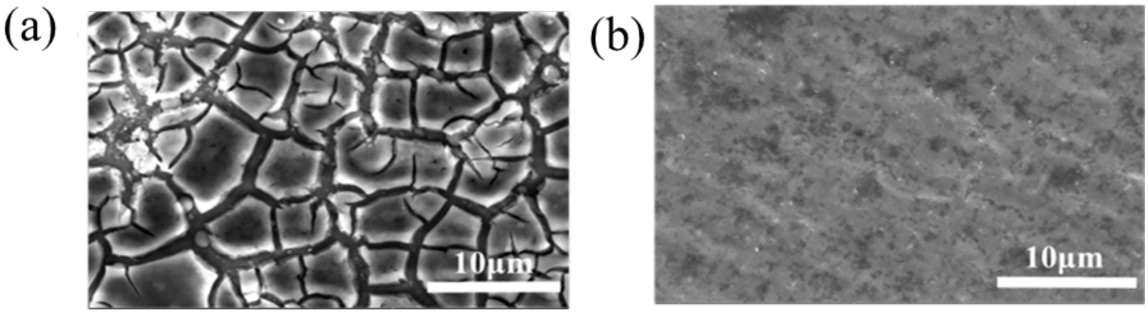

Figure S4 SEM images of the morphologies of Al foils after CVs in (a) the 1 M LiPF6 electrolyte and (b) the 4 M LiODFB-DME electrolyte.
